# Gigwa—Genotype investigator for genome-wide analyses

**DOI:** 10.1186/s13742-016-0131-8

**Published:** 2016-06-06

**Authors:** Guilhem Sempéré, Florian Philippe, Alexis Dereeper, Manuel Ruiz, Gautier Sarah, Pierre Larmande

**Affiliations:** UMR InterTryp (CIRAD), Campus International de Baillarguet, 34398, Montpellier, Cedex 5 France; South Green Bioinformatics Platform, 1000 Avenue Agropolis, 34934 Montpellier, Cedex 5 France; UMR DIADE (IRD), 911 Avenue Agropolis, 34934 Montpellier, Cedex 5 France; UMR IPME (IRD), 911 Avenue Agropolis, 34394 Montpellier, Cedex 5 France; UMR AGAP, CIRAD, 34398 Montpellier, Cedex 5 France; Institut de Biologie Computationnelle, Université de Montpellier, 860 Rue de St Priest, 34095 Montpellier, Cedex 5 France; Agrobiodiversity Research Area, International Center for Tropical Agriculture (CIAT), 6713 Cali, Colombia; INRA, UMR AGAP, 34398 Montpellier, Cedex 5 France; INRIA Zenith Team, LIRMM, 161 Rue Ada, 34095 Montpellier, Cedex 5 France

**Keywords:** Genomic variations, VCF, HapMap, NoSQL, MongoDB, SNP, INDEL, Web interface

## Abstract

**Background:**

Exploring the structure of genomes and analyzing their evolution is essential to understanding the ecological adaptation of organisms. However, with the large amounts of data being produced by next-generation sequencing, computational challenges arise in terms of storage, search, sharing, analysis and visualization. This is particularly true with regards to studies of genomic variation, which are currently lacking scalable and user-friendly data exploration solutions.

**Description:**

Here we present Gigwa, a web-based tool that provides an easy and intuitive way to explore large amounts of genotyping data by filtering it not only on the basis of variant features, including functional annotations, but also on genotype patterns. The data storage relies on MongoDB, which offers good scalability properties. Gigwa can handle multiple databases and may be deployed in either single- or multi-user mode. In addition, it provides a wide range of popular export formats.

**Conclusions:**

The Gigwa application is suitable for managing large amounts of genomic variation data. Its user-friendly web interface makes such processing widely accessible. It can either be simply deployed on a workstation or be used to provide a shared data portal for a given community of researchers.

**Electronic supplementary material:**

The online version of this article (doi:10.1186/s13742-016-0131-8) contains supplementary material, which is available to authorized users.

## Findings

### Background

With the advent of next-generation sequencing (NGS) technology, thousands of new genomes of both plant and animal organisms have recently become available. Whole exome and genome sequencing, genotyping-by-sequencing and restriction site-associated DNA sequencing (RADseq) are all becoming standard methods to detect single-nucleotide polymorphisms (SNPs) and insertions/deletions (indels), in order to identify causal mutations or study the associations between genetic variations and functional traits [1–4]. As a result, huge amounts of gene sequence variation data are accumulating in numerous species-oriented projects, such as 3000 rice genomes [5] or 1001 *Arabidopsis* genomes [6, 7]. In this context, the Variant Call Format (VCF) [8] has become a convenient and standard file format for storing variants identified by NGS approaches.

VCF files may contain information on tens of millions of variants, for thousands of individuals. Having to manage such significant volumes of data involves considerations of efficiency with regard to the following aspects:

1. Filtering features. Such data can be processed by applications like VCFtools [8], GATK [9], PyVCF [10], VariantAnnotation [11] or WhopGenome [12] to query, filter and extract expertized datasets for day-to-day research. However, these tools are limited to command line or programmatic application programming interfaces (APIs) targeted at experienced users, and are not suitable for non-bioinformaticians.
2. Storage performance. Working with flat files is not an optimal solution in cases where scientists need to establish comparisons across projects and/or take metadata into account. The use of relational databases is still widely prevalent within the range of more integrated approaches. However, such solutions have limitations when managing big data [13]. In computational environments with large amounts of heterogeneous data, the NoSQL database technology [14, 15] has emerged as an alternative to traditional relational database management systems. NoSQL refers to non-relational database management systems designed for large-scale data storage and massively parallel data processing. During the past 5 years, a number of bioinformatics projects have been developed based on NoSQL databases such as HBase [16, 17], Hadoop [18–20], Persevere [21], Cassandra [22] and CouchDB [23].
3. Sharing capabilities. This aspect is clearly best addressed by providing client/server-based applications, which enable multiple users to work on the same dataset without the need to replicate it for each user. There is, as yet, a considerable lack of web applications able to handle the potentially huge genotyping datasets that are emerging from mass genotyping projects, and which would enable biologists to easily access, query and analyze data online.
4. Graphical visualization. A number of solutions have been developed for the graphical visualization of genomic variation datasets. Some of these have been integrated into data portals associated with specific projects (e.g. OryzaGenome [24], SNP-Seek [25]) and are, therefore, only relevant to a particular community. Generic tools also exist (e.g. vcf.iobio [26, 27], JBrowse [28]) and may be built upon to create more versatile applications.

The Gigwa application, the name of which stands for ‘Genotype investigator for genome-wide analyses’, aims to take account of all of these aspects. It is a web-based, platform-independent solution that feeds a MongoDB [29] NoSQL database with VCF or HapMap files containing billions of genotypes, and provides a web interface to filter data in real time. In terms of visualization, the first version includes only an online density chart generator. However, Gigwa supplies the means to export filtered data in several popular formats, thus facilitating connectivity with many existing visualization engines.

### Application description

**Fig. 1:**
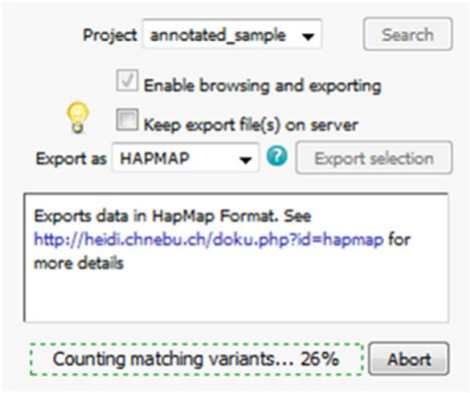
Action panel enabling project selection, progress indication, abort and export functionalities

A single instance of the Gigwa application is able to display data from multiple databases, which can be chosen from a drop-down menu at the top of the page. A database may syndicate any source of genotyping data as long as the variant positions are provided on the same reference assembly. Gigwa supports work on a single project at a time (although a project may be divided into several runs, in which case new data connected to existing individuals are seen as additional samples). Project selection may be changed from within the action panel (Fig. 1) that sits to the right-hand side of the screen. This panel also enables the launch of searches, toggles the availability of browsing and exporting functions (because limiting the initial approach to the counting of results saves time), configures and launches the export, checks the progress of ongoing operations, and can terminate them if required.

**Fig. 2:**
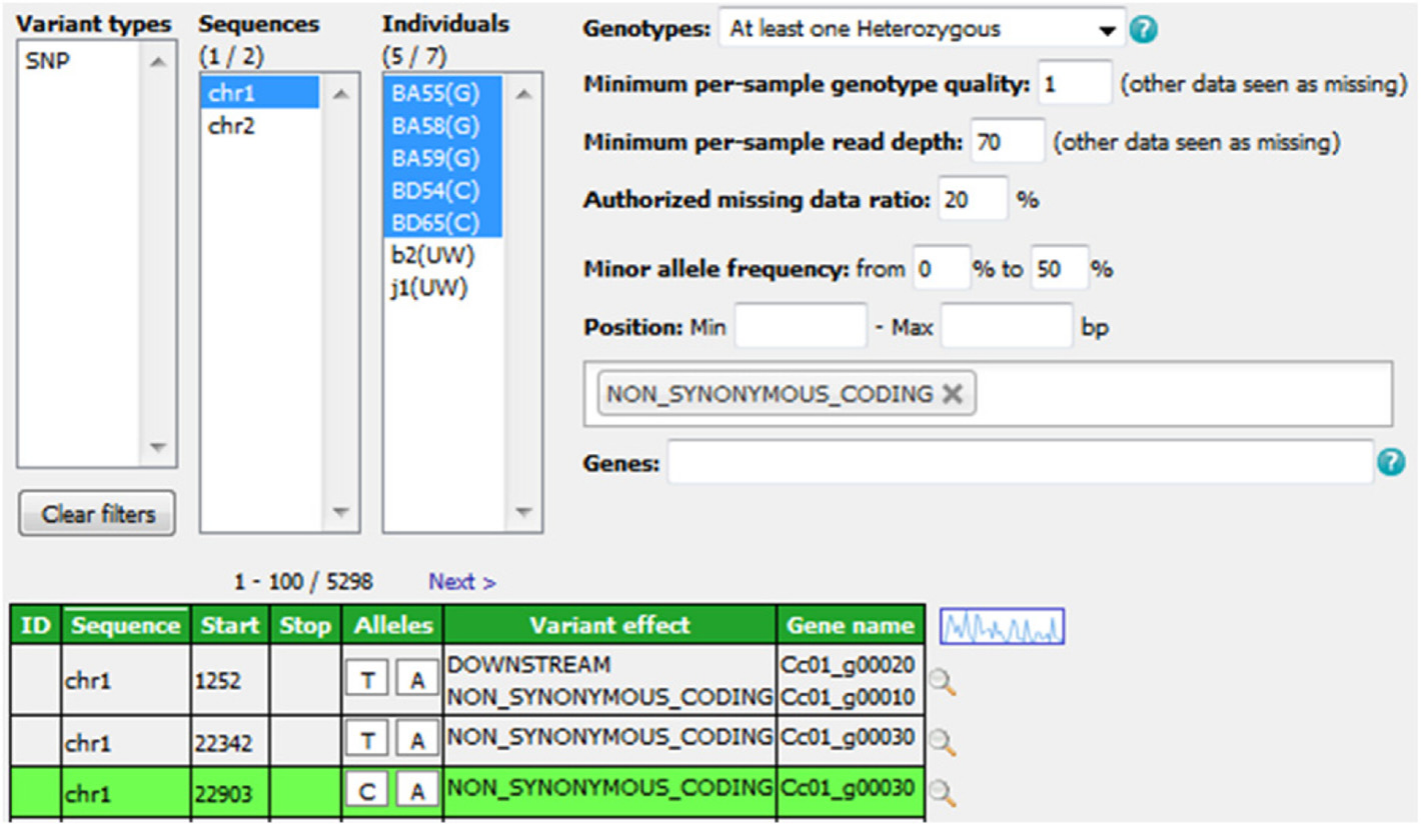
Variant-filtering interface, allowing search criteria definition and result browsing

The variant-filtering interface (top of Fig. 2) is both compact and intuitive. In the top-left corner of this panel, three lists allow multiple-item selection of variation types (e.g. SNPs, indels, structural variants), individuals and reference sequences. More specific filters can be incorporated to refine searches using combinations of the following parameters:

- ‘Genotypes’ - this filter makes it possible to retrieve only variant positions that respect a specified genotyping pattern when considering selected individuals. If no individuals are selected, the application takes them all into account. A dozen predefined options (e.g. all same, at least one heterozygous) are available, covering those cases that are most frequently meaningful.
- ‘Minimum per-sample genotype quality’ and ‘Minimum per-sample read depth’ - these individual-based filters may be used, in the case of data from VCF files, to set thresholds on the quality (GQ) and depth (DP) fields assigned to genotypes [30]. Individuals that do not meet these criteria are subsequently treated as missing data.
- ‘Authorized missing data ratio’ - this filter allows a maximum threshold of acceptable missing data among selected individuals to be defined. Its default value is 100 %, that is, accepting all data.
- ‘Minor allele frequency’ (MAF) - this filter retains only the variant positions for which the MAF calculated on selected individuals falls in the specified range (by default, 0–50 %). It is only applicable to bi-allelic markers.
- ‘Number of alleles’ - this filter allows specification of the number of known alleles the targeted variants are expected to have.
- ‘Position’ - this filter restricts the search to variants located in a given range of positions in relation to the reference.
- SnpEff widgets - these allow additional filtering on variant effects and gene names for data originating from VCF files that have been annotated with SnpEff [31]. The application automatically detects such additional data and is able to handle both types of annotation field, that is, ‘EFF’ (SnpEff versions prior to 4.1) and ‘ANN’ (SnpEff versions from 4.1 onwards).

**Fig 3:**
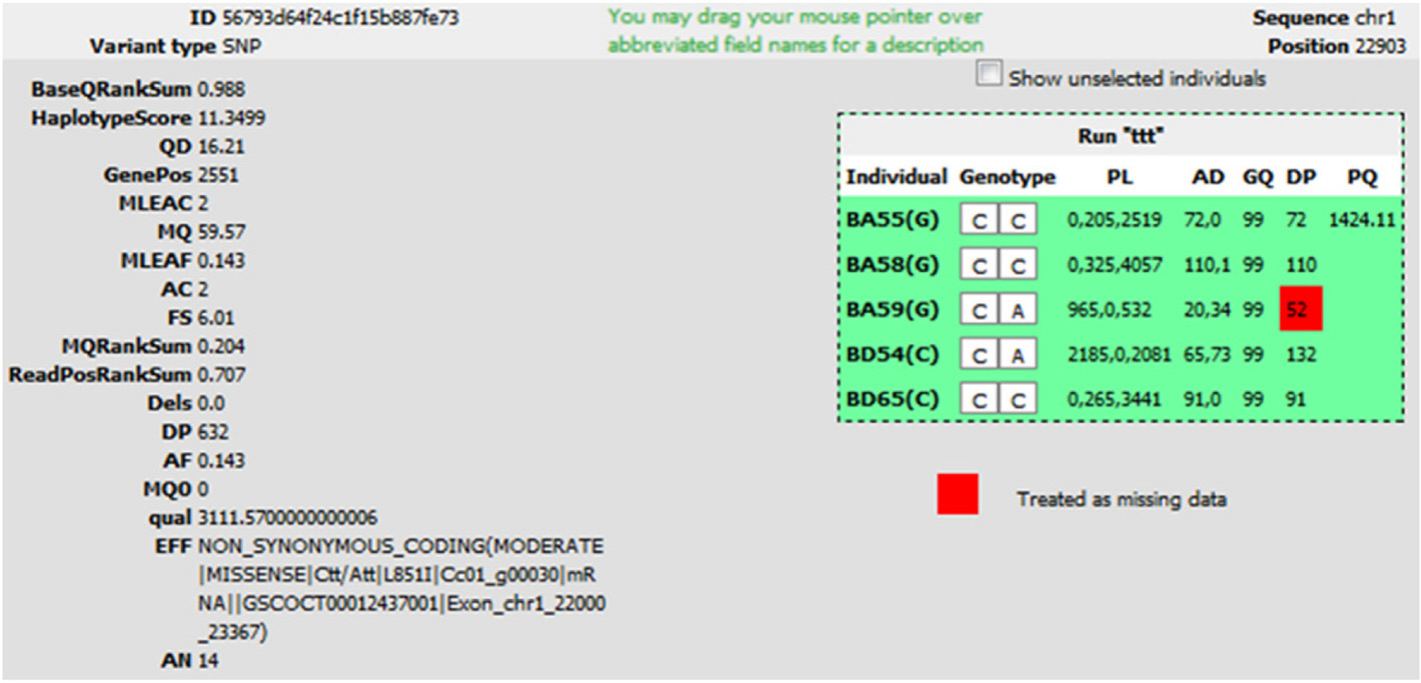
Variant detail dialogue, providing variant metadata and genotype-level information

Matching variants are displayed in paginated form (see bottom of Fig. 2) after application of the filters. Results are listed in a sortable table that provides the main attributes, namely ID (when provided in the input file), reference sequence, start and stop positions, alleles, variant effect and gene name (the latter two only being displayed if available). In addition, the user can focus on a specific position and display variant details, including selected individuals’ genotypes, using the magnifier at the end of each row. These details appear in a dialogue (Fig. 3) that, for each run in the selected project, provides:

- additional variant-level attributes or annotations (global attributes related to the variant), on the left of the screen;
- on the right of the screen, a box indicating each individual’s genotype, along with genotype-level attributes (e.g. depth, quality). A checkbox allows the display of genotypes for unselected individuals. Any GQ and DP values that are below specified thresholds, and have thus led to a genotype being considered as missing, are highlighted with a red background.

### Data export and visualization

The Gigwa application offers seven standardized formats (VCF, Eigenstrat, GFF3, BED, HapMap, DARwin and PLINK) in which to export filtered results in compressed files. Export is individual-based. Thus, if the data selection includes several samples that belong to the same individual, only one genotype per variant is exported. If these genotypes are inconsistent, the one most frequently found is selected. If there is no most frequently found genotype, one is picked at random.

Where data that originated from VCF files is being re-exported in the same format, the application takes phased genotypes into account: a procedure was implemented to maintain phasing information (i.e. haplotype estimation) in the database and recalculate it at export time, even if intermediate positions had been filtered out.

Exports can be directed either to the client computer or to a temporary URL on a web server, thus making the dataset instantly shareable, for example, with Galaxy [32]. Such links remain active for a week. In addition, when applied to the VCF format, this ‘export to URL’ feature provides the means for users to view selected variants in their genomic context in a running instance of the Integrative Genomics Viewer (IGV) [33].

The current selection may also be directed to an online, interactive, density chart viewer. The variant distribution of each sequence may then be observed, with the ability to filter on variation type, and these figures can also be exported in various file formats (i.e. PNG, JPEG, PDF and SVG).

### Technical insights

#### Third-party software involved

MongoDB [29] was chosen as the storage layer for several reasons: its complex query support, its scalability, its open-source nature and its proactive support community. The server application was developed in Java and takes advantage of several Spring Framework modules [34] (e.g. Spring Data). The client interface was designed using Java Server Pages (JSP) and jQuery [35]. Some import and export procedures make use of the SAMtools HTSJDK API v1.143 [36]. The density visualization tool was implemented using the HighCharts Javascript library [37].

#### Data structure

The data model for storing genotyping information, defined using Spring Data documents, is shared with the WIDDE application [21] and allows a single database to hold genotypes from multiple runs of multiple projects. This model is marker-oriented and mainly relies on two basic document types: *VariantData*, which embeds variant-level information (e.g. position, marker type); *VariantRunData*, which contains genotyping data along with possible metadata.

A collection named *taggedVariants* is not tied to a model object because its documents only contain variant IDs. However, it serves an important purpose by providing dividers (‘landmarks’) that partition the entire collection of variants into evenly sized chunks. These chunks are then used when querying directly on the *VariantRunData* collection (i.e. without a preliminary filter on variant features) to split the query into several sub-queries, which confers several advantages (see Querying strategy below).

Less significant model objects include *GenotypingProject*, which keeps track of elements used to rapidly build the interface (e.g. distinct lists of sequence names and variant types involved in the project), and *DBVCFHeader*, which simply stores the contents of headers for runs imported in VCF format.

#### Querying strategy

When the *Search* button is clicked, the values selected in the search-interface widgets are passed to the server application. They may then be used to count and/or browse matching variants.

The first time a given combination of filters is invoked, the *count* procedure is launched to establish the number of variants that match the combination. This result is then cached in a dedicated collection so that whenever a user subsequently repeats the same search, the result will be available instantly.

Once the *count* result has been displayed to the user, if the ‘Enable browsing and exporting’ box is checked, a second request is sent to the server, invoking the *find* procedure that eventually provides paginated, detailed variant information in the form of a comprehensive table.

In general, serving such requests (*count* or *find*) may be divided into two consecutive steps:

- a simple, preliminary query of variant features (variant type, sequence, start position), which is applied to indexed fields and therefore executes quickly;
- the main aggregation query, which is split into several partial queries aimed at running in simultaneous threads on evenly sized variant chunks of the *VariantRunData* collection. This technique not only improves performance, but also allows Gigwa to provide a progress indicator and the facility to terminate a run before it has finished. The method used for dividing the main query depends on whether or not a preliminary filter was executed beforehand. If it was, the application holds a subset of variant IDs as a consequence, which it uses to split the data using MongoDB’s *$in* operator in each sub-query. Otherwise, the contents of the *taggedVariants* collection are used, in conjunction with the *$lte* (less than or equal) and *$gt* (greater than) operators, to define the limits of each sub-query’s chunk.

### Summary of features

Gigwa’s value resides in the following features:

- Support for large genotyping files with up to several million variants
- Responsive queries even in the case of a local deployment
- Intuitive graphical user interface allowing the definition of precise queries in a few clicks
- Filtering on functional annotations
- Ability to abort running queries
- Display of query progress
- Support of multiple data sources for a single instance
- A multi-user mode which enables both public and private access to databases to be defined
- Support for incremental data loading
- Support for seven different export formats
- Easy connection with IGV for integration within a consistent genomic context
- No loss of phasing information when provided (VCF format only)
- Support for haploid, diploid and polyploid data
- Online variant density viewing.

The Gigwa application therefore represents a very efficient, versatile and user-friendly tool for users with standard levels of expertise in web navigation to explore large amounts of genotyping data, identify variants of interest and export subsets of data in a convenient format for further analysis. We believe that its large panel of undoubtedly useful features will make Gigwa an essential tool in the increasingly complex field of genomics.

### Benchmarking

In order to assess Gigwa’s performance, we conducted benchmarks against comparable applications.

#### Hardware used

All tests were run on an IBM dx360 M2 server with:

- two quad-core CPUs (Core-i7 L5520 at 2.26 GHz);
- 36 GB RAM (DDR3 at 1333 MHz);
- 250-GB SATA2 hard drive.

#### Dataset selection

As our base dataset, we chose to use the CoreSNP dataset from the 3000 Rice Genomes Project [5, 38], which at the time of download (v2.1) contained genotypes for 3000 individuals on 365,710 SNPs. This dataset was first converted to a 4.09-GB VCF file using VCFtools, from which three progressively smaller datasets were then generated by successively dividing the number of variants by ten, i.e. resulting in datasets of 36,571, 3658 and 366 SNPs, respectively.

#### Benchmark comparisons

We considered it appropriate to compare Gigwa’s performance with that of:

1. VCFtools (v0.1.13) [12];
2. A MySQL (v5.6.28) [39] implementation of a standard relational database model with indexes on appropriate fields. Corresponding queries were implemented as stored procedures, and both these and the database schema are provided as supplementary material within the supporting data.

In addition, the opportunity was taken to evaluate the relative performance of the currently available storage solutions offered by MongoDB v3.0.6, i.e. the newly introduced WiredTiger (WT) storage engine, configured with three different compression levels (none, *snappy* and *zlib*), and the original MMapv1 storage engine.

Therefore, each of the benchmarking plots generated contains six series: VCFtools, MySQL, Gigwa-MMapv1, Gigwa-WT-none, Gigwa-WT-snappy and Gigwa-WT-zlib. MongoDB queries were launched via the Gigwa interface because, internally, the application splits them into a number of partial, concurrent queries.

#### Benchmark queries

Two kinds of queries that we considered representative were executed as benchmarks on each dataset for each tool:

- Location-based query: a query counting variants located in a defined region of a chromosome (chromosome 3, 1 Mbp to 5 Mbp).
- Genotype-based query: a query counting variants exhibiting a given MAF range (10 to 30 %) on the first 2000 individuals (out of 3000).

All benchmarks were executed three times, except for the MAF queries in Gigwa where the different MongoDB configurations gave response times showing high degrees of heterogeneity. In order to establish more distinction between them, these benchmarks were therefore executed 12 times. In all cases, average response times were calculated and then reported through graphical plots. The caching system implemented in Gigwa was disabled for the duration of the benchmarking.

#### Benchmark results

**Fig. 4:**
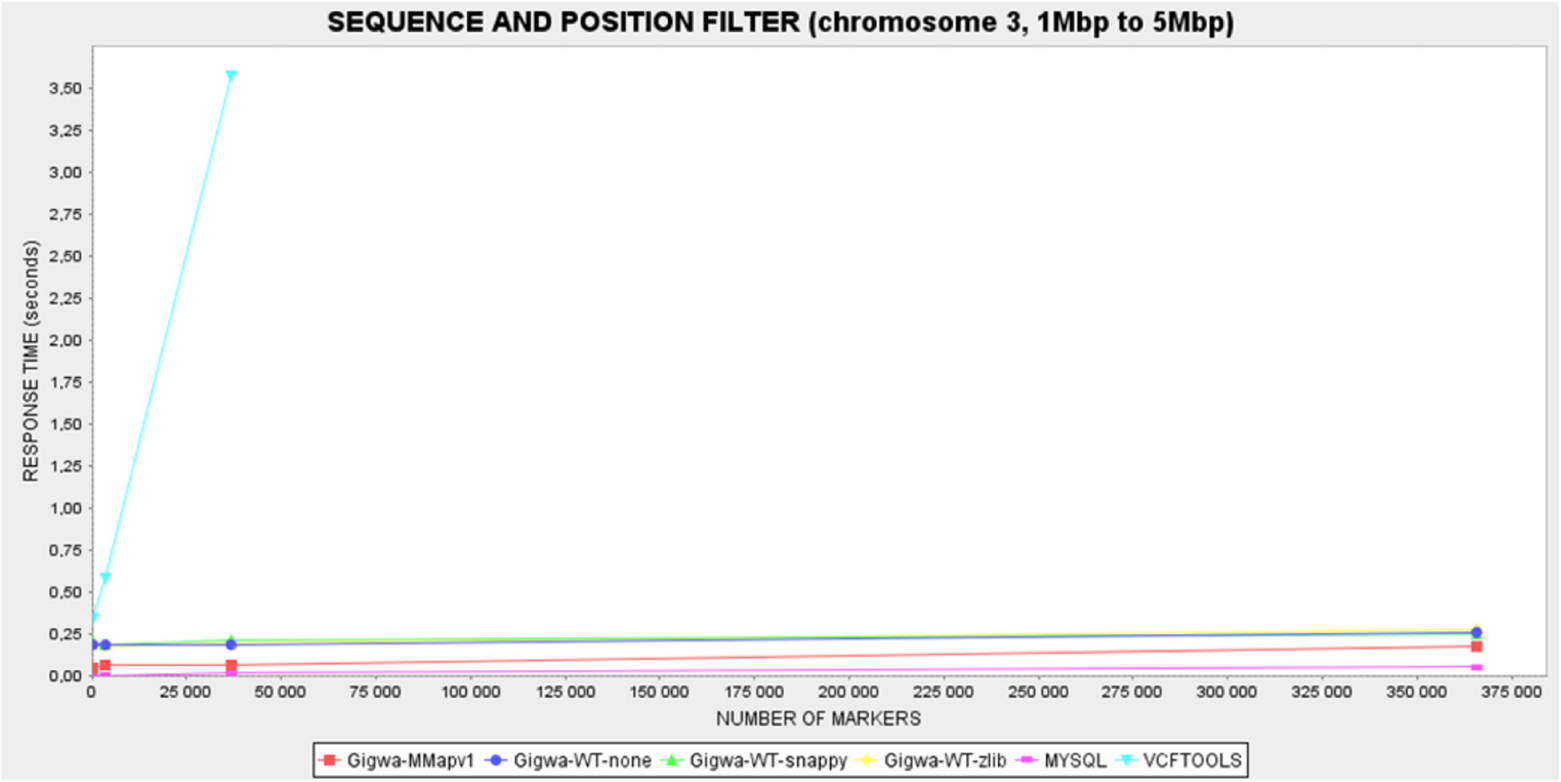
Response-time plot by tool for first benchmarked query (location-based filter). VCFtools is by far the slowest option

In the case of the location-based filter benchmark (Fig. 4), the MySQL solution was the fastest, with response times that were negligible on the smallest dataset, and never more than 0.05 s on the largest. In comparison, Gigwa queries were less responsive but still remained fairly fast, never taking more than 0.3 s on the largest dataset. However, VCFtools proved so much slower than all the other alternatives benchmarked that we had to exclude its last record for the plot to remain readable. This difference can be explained by the fact that database engines typically take advantage of pre-built indexes that lead directly to results, whereas VCFtools has to scan the entire file. Relational databases are usually the most efficient for this kind of simple query because their indexing mechanisms have been optimized over decades.

**Fig. 5:**
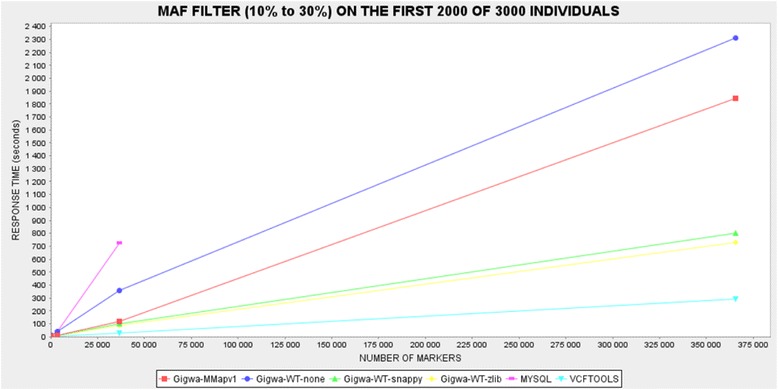
Response-time plot by tool for second benchmarked query (MAF filter). MySQL is by far the slowest option

In the case of the MAF filter benchmark (Fig. 5), the fastest solution was VCFtools, followed by the two most compressed MongoDB databases (Gigwa-WT-zlib and Gigwa-WT-snappy), and then by the two least compressed MongoDB databases (Gigwa-MMapv1 and Gigwa-WT-none). The MySQL engine performed so poorly here that it was considered unnecessary to run the longest query on it. In practice, the type of analysis involved in this particular benchmark requires that all stored positions be scanned. VCFtools excels here because it is a C++ program working on flat files, which means that the time needed to access each record is negligible, whereas database engines need to obtain/deflate objects before manipulating them. In contrast to the situation seen in the first filter benchmark, a significant difference in performance emerged here between the various storage solutions offered by MongoDB. There is more room in this benchmark for performance distinctions because memory consumption becomes more crucial when executing a multi-step aggregation pipeline rather than a simple index count. WiredTiger applies compression to indexes, which leaves more memory available for other tasks, thus increasing performance. In addition, WiredTiger is known to perform better than MMapv1 on multi-threaded queries, which are being used by Gigwa.

Thus, Gigwa configured with WiredTiger-snappy (or WiredTiger-zlib in the case of constraints on disk space) appears to be an excellent compromise, being the only solution that responds in a reasonable time to both kinds of query. Furthermore, although it was beyond the scope of this benchmark, we should mention that the greatly reduced storage space required by both WiredTiger-snappy and WiredTiger-zlib, when compared with that required by MMapv1, provides an additional justification for choosing WiredTiger in most cases.

## Conclusions

We developed Gigwa to manage large genomic variation data derived from NGS analyses or high-throughput genotyping. The application aims to provide a user-friendly web interface that makes real-time filtering of such data, based on variant features and individuals’ genotypes, widely accessible. Gigwa can be deployed either in single-user mode or in multi-user mode, with credentials and permissions allowing fine-grained control of access to connected databases.

We ran benchmarks on two kinds of queries - variant-oriented and genotype-oriented - to compare Gigwa’s performance with that of both VCFtools and a standard MySQL model. Each of these latter tools performed best in one benchmark but by far the worst in the other. Gigwa, when configured with the WiredTiger storage engine and either the *snappy* or *zlib* compression level, appeared as an excellent compromise, performing almost as well as the best solution in both benchmarks.

Future versions of Gigwa will include a RESTful API to allow external applications to interact with Gigwa and query data in a standardized manner, as well as additional visualization tools and a Docker [40] package aimed at distributing the tool as a solution capable of functioning in platform-as-a-service (PaaS) [41] mode. Further benchmarks will be conducted to evaluate the application’s performance in a distributed environment using MongoDB’s sharding functionality.

## Availability and requirements

- **Project name:** Gigwa
- **Project home page:**http://www.southgreen.fr/content/gigwa
- **Operating system(s):** Platform-independent
- **Programming language:** Java & MongoDB
- **Requirements:** Java 7 or higher, Tomcat 7 or higher, MongoDB 3 or higher
- **License:** GNU GPLv3
- **Restrictions to use for non-academics:** None

## Additional file

Additional file 1: Gigwa, Genotype investigator for genome-wide analyses. Provides the MySQL scripts used for benchmarking and guidelines on how to import data into Gigwa and configure its access for existing users. (DOCX 95 kb)
