# Erratum to: Gigwa-Genotype investigator for genome-wide analyses

**DOI:** 10.1186/s13742-016-0153-2

**Published:** 2016-11-02

**Authors:** Guilhem Sempéré, Florian Philippe, Alexis Dereeper, Manuel Ruiz, Gautier Sarah, Pierre Larmande

**Affiliations:** 1 UMR InterTryp (CIRAD), Campus International de Baillarguet, 34398 Montpellier, Cedex 5, France; 2 South Green Bioinformatics Platform, 1000 Avenue Agropolis, 34934 Montpellier, Cedex 5, France; 3 UMR DIADE (IRD), 911 Avenue Agropolis, 34934 Montpellier, Cedex 5, France; 4 UMR IPME (IRD), 911 Avenue Agropolis, 34394 Montpellier, Cedex 5, France; 5 UMR AGAP, CIRAD, 34398 Montpellier, Cedex 5, France; 6 Institut de Biologie Computationnelle, Université de Montpellier, 860 Rue de St Priest, 34095 Montpellier, Cedex 5, France; 7 Agrobiodiversity Research Area, International Center for Tropical Agriculture (CIAT), 6713, Cali, Colombia; 8 INRA, UMR AGAP, 34398, Montpellier, Cedex 5, France; 9 INRIA Zenith Team, LIRMM, 161 Rue Ada, 34095 Montpellier, Cedex 5, France

## Erratum

The original version of the article ‘Gigwa-Genotype investigator for genome-wide analyses. GigaScience 2016 5:25’ [1] unfortunately contained an error.

In the Technical insights - Data structure section, the mentioned WIDDE application actually refers to “WIDDE: a Web-Interfaced next generation database for genetic diversity exploration, with a first application in cattle. BMC Genomics 2015 16:940” [2], not to “Knowledge engineering tools for reasoning with scientific observations and interpretations: a neural connectivity use case. BMC Bioinf. 2011;12(1):351”.

## References

[1] Sempéré G, Philippe F, Dereeper A, Ruiz M, Sarah G, Larmande P (2016). Gigwa-Genotype investigator for genome-wide analyses. Gigascience [Internet].

[2] Sempéré G, Moazami-Goudarzi K, Eggen A, Laloë D, Gautier M, Flori L (2015). WIDDE: a Web-Interfaced next generation database for genetic diversity exploration, with a first application in cattle. BMC Genomics [Internet].

