# Supplementary material for: Gigwa—Genotype investigator for genome-wide analyses
Source: Gigascience. 2016 Jun 6;5:25. doi: 10.1186/s13742-016-0131-8 (PMC4897896; doi:10.1186/s13742-016-0131-8)
Supplement: Additional file 1: — Gigwa, Genotype investigator for genome-wide analyses. Provides the MySQL scripts used for benchmarking and guidelines on how to import data into Gigwa and configure its access for existing users. (DOCX 95 kb) [file 13742_2016_131_MOESM1_ESM.docx]

**Supplementary Data: Gigwa, Genotype Investigator for Genome Wide Analyses**

1. **VCFtools benchmark material**
   1. **Data files**

VCF files for the 4 benchmarked datasets are available at

https://<GIGADB_URL>/DATA/benchmarks/v1/

N.B.: The full.vcf file contains 365710 variants

- 1. **VCFtools filter scripts**

All 8 scripts (2 filters * 4 datasets) may be found at

[https:// <GIGADB_URL>/DATA/benchmarks/v1/VCFtools/](https://github.com/SouthGreenPlatform/Gigwa/DATA/benchmarks/v1/VCFtools/)

1. **MySQL scripts used for benchmarking**
   1. **Schema creation script**

The few next lines define the structure of a standard relational model storing genotyping data:

CREATE TABLE `variants` (`id` VARCHAR(45) NOT NULL, `chromosome` VARCHAR(45) NULL, `position` INT NULL, PRIMARY KEY (`id`));

CREATE TABLE `genotypingdata` (`variant_id` VARCHAR(45) NOT NULL, `individual` VARCHAR(45) NULL, `genotype` VARCHAR(102) NULL);

ALTER TABLE `variants` ADD INDEX `chromosome` (`chromosome` ASC);

Full scripts for creating the benchmark databases and import data into them (mysql_366var.zip, mysql_3658var.zip, mysql_36571var.zip) are provided at

https:// <GIGADB_URL>/DATA/benchmarks/v1/MySQL/

As stated in the article, the “full” dataset was not converted to MySQL because of performance concerns that made it irrelevant.

- 1. **Position filter stored procedure script**

This script creates a stored procedure that applies a position filter on the selected database:

DELIMITER $

CREATE PROCEDURE `chrompos`(chr SMALLINT, start BIGINT, stop BIGINT)

BEGIN

select count(distinct variants.position) from variants where variants.chromosome=chr and variants.position>=start and variants.position<=stop ;

END$

DELIMITER ;

It is provided as position_filter.sql in the last mentioned location.

- 1. **MAF filter stored procedure script**

This script is long because it contains a list of 2000 individual names. It is provided as maf_filter.sql in the last mentioned location.

1. **Detailed benchmark results**
   1. **Position filter results**

| **DATASETS** | **366 MARKERS** | **3658 MARKERS** | **36571 MARKERS** | **365710 MARKERS** |
| --- | --- | --- | --- | --- |
|  |  |  |  |  |
| **AVERAGE RESPONSE TIME**  **for VCFTOOLS (sec)** | **0.336666667** | **0.581666667** | **3.57** | **64.96133333** |
|  | 0.04 | 0.62 | 2.61 | 66.08 |
|  | 0.04 | 0.56 | 2.61 | 62.22 |
|  | 0.93 | 0.565 | 5.49 | 66.584 |
|  |  |  |  |  |
| **AVERAGE RESPONSE TIME**  **for Gigwa-MMapv1 (sec)** | **0.042** | **0.059** | **0.063** | **0.172** |
|  | 0.048 | 0.055 | 0.081 | 0.307 |
|  | 0.054 | 0.065 | 0.054 | 0.103 |
|  | 0.024 | 0.057 | 0.054 | 0.106 |
|  |  |  |  |  |
| **AVERAGE RESPONSE TIME**  **for Gigwa-WT-none (sec)** | **0.185** | **0.182666667** | **0.185666667** | **0.26** |
|  | 0.19 | 0.183 | 0.181 | 0.264 |
|  | 0.191 | 0.174 | 0.188 | 0.256 |
|  | 0.174 | 0.191 | 0.188 | 0.26 |
|  |  |  |  |  |
| **AVERAGE RESPONSE TIME**  **for Gigwa-WT-snappy (sec)** | **0.201333333** | **0.184333333** | **0.208** | **0.25** |
|  | 0.19 | 0.174 | 0.22 | 0.28 |
|  | 0.174 | 0.19 | 0.181 | 0.181 |
|  | 0.24 | 0.189 | 0.223 | 0.289 |
|  |  |  |  |  |
| **AVERAGE RESPONSE TIME**  **for Gigwa-WT-zlib (sec)** | **0.184333333** | **0.176666667** | **0.191666667** | **0.272666667** |
|  | 0.181 | 0.174 | 0.215 | 0.298 |
|  | 0.199 | 0.173 | 0.189 | 0.264 |
|  | 0.173 | 0.183 | 0.171 | 0.256 |
|  |  |  |  |  |
| **AVERAGE RESPONSE TIME**  **for MYSQL (sec)** | **0** | **0.001** | **0.015333333** | **0.05** |
|  | 0 | 0.001 | 0.006 | 0.05 |
|  | 0 | 0.001 | 0.02 | 0.05 |
|  | 0 | 0.001 | 0.02 | 0.05 |
|  |  |  |  |  |
| **FILTER RESULT** | 3 | 28 | 285 | 2850 |

- 1. **MAF filter results**

| **DATASETS** | **366 MARKERS** | **3658 MARKERS** | **36571 MARKERS** | **365710 MARKERS** |
| --- | --- | --- | --- | --- |
|  |  |  |  |  |
| **AVERAGE RESPONSE TIME**  **for VCFTOOLS (sec)** | **0.379** | **3.057666667** | **29.06666667** | **293.693** |
|  | 0.391 | 2.927 | 29.278 | 295.75 |
|  | 0.404 | 3.061 | 29.576 | 292.282 |
|  | 0.342 | 3.185 | 28.346 | 293.047 |
|  |  |  |  |  |
| **AVERAGE RESPONSE TIME**  **for Gigwa-MMapv1 (sec)** | **4.779166667** | **13.15633333** | **119.5165833** | **1845.203833** |
|  | 1.831 | 3.894 | 122.805 | 1472.048 |
|  | 3.543 | 13.982 | 95.749 | 1925.836 |
|  | 3.418 | 13.975 | 123.914 | 3795.527 |
|  | 3.924 | 14.056 | 122.685 | 1592.977 |
|  | 2.755 | 13.72 | 123.645 | 1593.481 |
|  | 5.404 | 13.32 | 115.285 | 1854.207 |
|  | 3.965 | 13.871 | 121.954 | 1589.176 |
|  | 8.04 | 16.938 | 134.409 | 1601.72 |
|  | 8.402 | 13.45 | 114.032 | 1812.467 |
|  | 2.723 | 13.631 | 123.147 | 1588.832 |
|  | 6.064 | 13.379 | 110.951 | 1728.752 |
|  | 7.281 | 13.66 | 125.623 | 1587.423 |
|  |  |  |  |  |
| **AVERAGE RESPONSE TIME**  **for Gigwa-WT-none (sec)** | **12.30833333** | **40.88166667** | **355.6471667** | **2309.090833** |
|  | 15.201 | 64.589 | 439.857 | 2216.084 |
|  | 18.393 | 36.323 | 330.875 | 2461.907 |
|  | 25.689 | 66.788 | 716.57 | 3423.403 |
|  | 3.134 | 20.882 | 203.967 | 2017.643 |
|  | 3.184 | 21.374 | 206.433 | 2024.618 |
|  | 22.356 | 81.383 | 505.521 | 2260.308 |
|  | 3.309 | 21.499 | 202.383 | 2009.37 |
|  | 3.042 | 21.299 | 204.908 | 2021.633 |
|  | 23.554 | 56.447 | 556.222 | 2872.966 |
|  | 3.242 | 21.182 | 204.684 | 1993.779 |
|  | 23.47 | 57.323 | 491.988 | 2407.502 |
|  | 3.126 | 21.491 | 204.358 | 1999.877 |
|  |  |  |  |  |
| **AVERAGE RESPONSE TIME**  **for Gigwa-WT-snappy (sec)** | **3.626083333** | **11.73441667** | **101.2076667** | **799.2039167** |
|  | 1.934 | 9.692 | 88.822 | 665.348 |
|  | 3.059 | 9.916 | 88.139 | 629.849 |
|  | 14.385 | 21.032 | 123.014 | 528.306 |
|  | 2.6 | 11.5 | 110.523 | 1022.571 |
|  | 2.417 | 11.2 | 95.781 | 853.915 |
|  | 3.283 | 10.7 | 88.049 | 542.617 |
|  | 2.434 | 11.166 | 103.356 | 1007.83 |
|  | 2.575 | 10.899 | 104.105 | 1007.938 |
|  | 3.4 | 11.458 | 103.53 | 626.016 |
|  | 2.476 | 10.975 | 104.463 | 1007.363 |
|  | 2.633 | 11.583 | 100.755 | 691.789 |
|  | 2.317 | 10.692 | 103.955 | 1006.905 |
|  |  |  |  |  |
| **AVERAGE RESPONSE TIME**  **for Gigwa-WT-zlib (sec)** | **2.458083333** | **9.820666667** | **86.66883333** | **731.2119167** |
|  | 2 | 9.8 | 77.939 | 434.746 |
|  | 2.775 | 10.691 | 86.656 | 652.19 |
|  | 2.284 | 9.857 | 87.939 | 592.442 |
|  | 2.268 | 10.25 | 91.864 | 869.5 |
|  | 2.25 | 9.65 | 85.572 | 775.403 |
|  | 3.884 | 9.417 | 83.397 | 685.489 |
|  | 2.259 | 9.517 | 82.489 | 870.459 |
|  | 2.317 | 9.875 | 90.331 | 857.376 |
|  | 2.817 | 9.817 | 85.105 | 660.981 |
|  | 2.542 | 10.016 | 90.764 | 862.834 |
|  | 2.3 | 9.1 | 87.106 | 646.856 |
|  | 1.801 | 9.858 | 90.864 | 866.267 |
|  |  |  |  |  |
| **AVERAGE RESPONSE TIME**  **for MYSQL (sec)** | **2.186666667** | **40.22333333** | **724.6933333** |  |
|  | 2.21 | 40.32 | 718.28 |  |
|  | 2.18 | 40.16 | 670.61 |  |
|  | 2.17 | 40.19 | 785.19 |  |
|  |  |  |  |  |
| **FILTER RESULT** | 71 | 666 | 7007 | 70905 |

1. **GIGWA administration interface**
   1. **Data submission**

**
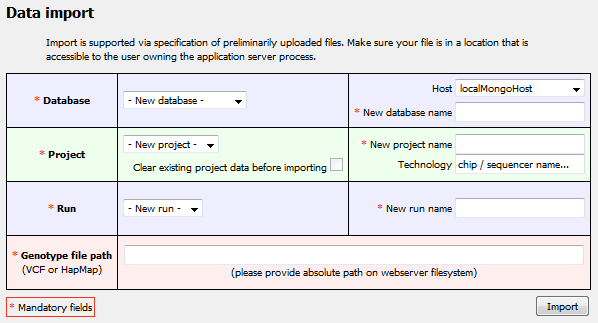
**

The standard process to import data is (1) uploading the data, (2) setting up the database, (3) configuring user privileges.

Uploading the data

Since import files may weigh several gigabytes, Gigwa does not provide any http upload feature. The administrator is therefore supposed to have means (FTP, SCP…) to place import files into the web server (the one running Tomcat), at a location where the user running the Tomcat process has read access.

Setting up the database

A single instance of Gigwa is able to work on several datasources (or modules). A datasource consists technically in a MongoDB host plus a database name, but is exposed to users as a single string (module name). The data import interface allows adding the contents of a VCF or HapMap file to a new or an existing module (see figure 1 above) by filling the contents of the following rows:

- The first row lets the administrator select an existing module or define a new one, by specifying a host and a database name.
- A given module may contain data from several projects. The condition for this is that all projects in a same module are based on the same reference assembly. However, Gigwa will only let users work on a single project at a time.
- A given project may contain several runs (in fact an import file corresponds to a run). This feature can be useful in situations where you obtained separate files for various populations, and intend to use Gigwa to compare their contents.
- Genotype file path expects the location of the import file on the web server. Remember it has to be readable by the user running Tomcat process (i.e. grant access to the file).

Assuming entered values are valid, clicking the “import” button launches the actual import into the selected database, while keeping the administrator informed of how many lines have been treated so far. Once finished, a link is provided to visualize the new data. At that stage, if the datasource is new, it is created private, and no user apart from the administrator has access to it.

Configuring user privileges

The last step consists, again in the case of a newly created module, in defining its level of privacy and/or granting users privileges to it. This is achieved by amending by hand some simple (text) configuration files, then reloading the application using the blue button provided in the web interface.

For more details about this part, we encourage readers to refer to the administration guide available at:

<https://github.com/SouthGreenPlatform/gigwa/blob/master/README.md#administration-guide>

- 1. **Security

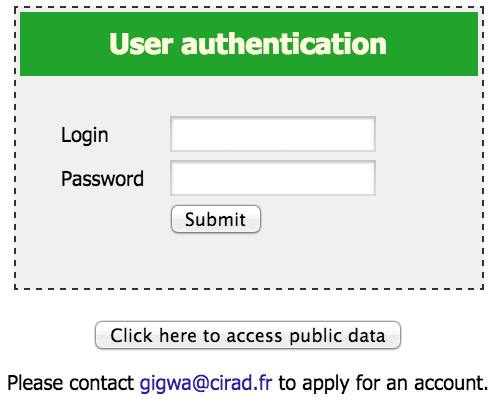
**

By default, Gigwa runs in a non-secure mode, in which anyone knowing its base URL can access all connected databases. This is good enough for some situations (installation on a local computer, or when all its data is public). It supports, however, a more advanced configuration, based on Spring-Security, which allows declaring lists of users and roles, thus defining who can access what.

Enabling multi-user mode has the following consequences: access to modules other than the public ones requires logging in using credentials defined by hand in a properties file. This file allows to create credentials for administrators (who can see all data, and import new data), or simple users, who need to be granted specific permissions to view private modules’ data.

Precise details about how to setup multi-user mode are provided in the administration guide available at <https://github.com/SouthGreenPlatform/gigwa>
